# Role of Montelukast as Monotherapy in Improving Quality of Life of an Asthmatic Patient in Pakistan

**DOI:** 10.7759/cureus.10841

**Published:** 2020-10-07

**Authors:** Ankeeta Kumari, Mishal Ejaz, Khurram Anis, Sumeet Kumar, Besham Kumar, Sidra Memon

**Affiliations:** 1 Medicine, Liaquat University of Medical and Health Sciences, Jamshoro, PAK; 2 Internal Medicine, Ziauddin University, Karachi, PAK; 3 Gastroenterology/Hepatology, Pakistan Kidney and Liver Institute, Lahore, PAK; 4 Internal Medicine, Chandka Medical College Hospital, Larkana, PAK; 5 Internal Medicine, Jinnah Postgraduate Medical Centre, Karachi, PAK; 6 Internal Medicine, Jinnah Sindh Medical University, Karachi, PAK

**Keywords:** montelukast, pakistan, quality of life

## Abstract

Introduction: Uncontrolled and inadequately managed asthma substantially reduces Quality of Life (QOL) and can lead to premature death. The aim of this study is to understand the role of montelukast in improving quality of life in asthmatic patients by comparing it with placebo.

Methods: This prospective, single-arm, interventional study was conducted from September 2019 to February 2020 in the pulmonology outpatient department of a tertiary care hospital in Pakistan. All patients were prescribed montelukast (10 mg once daily).

Results: At day 28, participants had a higher score on the Asthma Quality of Life Questionnaire-Standard (AQLQ-S) overall and in all sub-domains compared to day 0. The improvement was significant overall and for the sub-domains of symptoms, activity limitations, and environmental stimuli.

Conclusion: Montelukast has an effective role in asthma control as well as in improving the quality of life in the Pakistani population.

## Introduction

Asthma is characterized by an airway reaction leading to reversible airflow obstruction in association with airway hyper-responsiveness (AHR) and airway inflammation [1]. Asthma is quite common worldwide affecting approximately 339 million people, and according to studies, it is estimated to affect 100 million more people by the year 2025 [2]. When comparing geographical and demographic data, it is variable. Studies have suggested that high-income countries of the world have more people suffering from asthma than in average or low-income countries [3]. The report on global burden of asthma estimated the prevalence of asthma in Pakistan to be 4.3% [4]. Despite being quite common, it is not adequately being managed, leading to low Quality of Life (QOL) and early death. Asthma is rated as the 16th cause of Disability Adjusted Life Years (DALYs). Asthma also leads to increased burden of disease, as it ranks 28th among other causes [2].

Management of asthma is crucial. Inhaled corticosteroids (ICS) prove to produce promising results, however leukotriene receptor antagonists (LTRAs) are becoming more and more popular these days due to feasibility of use. LTRAs in contrast to ICS do not predispose the patient to long-term adverse effects [5]. LTRAs have been recommended by Global Initiative for Asthma (GINA) guidelines to be used as a single agent for asthma, can be used as an add-on or substitute to ICS increasing doses, or long-acting β2-agonist (LABA) [6].

In Pakistan, fewer studies have been conducted that study the role of montelukast in the management of asthma. Hence our aim is to study its role to help pulmonologists manage asthma effectively by reducing the morbidity and mortality of the disease.

## Materials and methods

This prospective, single-arm, interventional study was conducted from September 2019 to February 2020 in the pulmonology outpatient department of a tertiary care hospital in Pakistan. Both male and female asthmatic patients who were older than 15 years were enrolled in this study. Patients who presented with acute exacerbations of asthma and those who had previously taken montelukast were excluded from the study. All patients were given montelukast (10 mg once daily). Compliant patients were defined as those who took montelukast for at least 21 days. The patients were instructed to bring their strips on follow-up to ensure compliance.

A total of 156 participants were enrolled and were followed for four weeks. Patients who weren’t followed-up were not included in the analysis. A self-structured questionnaire was used to record the participant’s age, gender, duration of asthma, and symptoms. Quality of life was evaluated using the Asthma Quality of Life Questionnaire-Standard (AQLQ-S) on day 0 and day 60. AQLQ-S has 32 items assessing four domains: symptoms, activity, emotions, and environment control. A higher score means a higher quality of life [7]. 

Data were processed through and analyzed using SPSS for Windows version 23.0 (IBM Corp., Armonk, NY, USA). AQLQ-S mean and standard deviation (SD) were calculated for continuous variables such as age and AQLQ-S. Frequency and percentage were calculated for categorical variables including gender, age, duration of asthma, and symptoms. A comparison was done for scores of AQLQ-S on days 0 and 60 using dependent T-test. P-values of less than 0.05 meant there is a difference between AQLQ-S score on day 0 and day 60 and that the null hypothesis is void.

## Results

At the start of the study, 156 asthmatic patients were included. During the study, one (0.6%) patient stopped taking montelukast due to an adverse event and 21 (13.4%) participants were not followed-up. The study was completed by 134 (85.9%) participants, who were included in the final analysis. All these participants were compliant. The mean age of participants included in the analysis was 22 ± 8 years, of which 68 (50.7%) participants were men and 66 (49.3%) were women. Their disease characteristics are shown in Table 1

**Table 1:** Patient Characteristics

Patient Characteristics	Frequency (%) (n=134)
Duration of Asthma	
<1 year	15 (11.1%)
1-3 years	47 (35.0%)
3-6 years	38 (25.8%)
≥ 7 years	34 (25.3%)
Symptoms	
Cough	105 (78.3%)
Wheezing	99 (73.8%)
Chest Tightness	88 (65.6%)
Shortness of Breath	97 (72.3%)

At day 28, participants had a higher score on the Asthma Quality of Life Questionnaire-Standard (AQLQ-S) overall and in all sub-domains compared to day 0. The improvement was significant overall and for the sub-domains of symptoms, activity limitations, and environmental stimuli (Table 2).

**Table 2:** AQLQ-S at Day 0 and Day 28 Abbreviations: AQLQ-S, Asthma Quality of Life Questionnaire – Standard; SD, standard deviation; *Dependent sample T-test applied between groups; p value < 0.05 significant.

AQLQ-S	At Day 0 (Mean ± SD)	At Day 28 (Mean ± SD)	Difference	P value*
Overall	4.12 ± 0.62	4.89 ± 0.89	0.77	<0.0001*
Symptoms	3.79 ± 0.75	4.18 ± 0.37	0.39	<0.0001*
Activity Limitation	4.41 ± 0.99	5.11 ± 0.93	0.70	<0.0001*
Emotional Function	4.42 ± 1.20	4.52 ± 1.13	0.10	0.48
Environmental Function	4.59 ± 1.03	5.38 ± 1.21	0.79	<0.0001*

## Discussion

Montelukast is a leukotriene receptor antagonist. It reduces leukotriene D4 (LTD4) levels in the respiratory tract by competetively binding to the cysteinyl leukotriene receptor (CysLT1). LTD4 is a potent broncho-constrictor and CysLT1 attracts inflammatory cells. Normally, the binding of CysLT1 with LTD4 leads to allergic and hypersensitivity reactions, therefore montelukast plays a vital role in the management of asthmatic patients [8].

In this study, there was significant improvement in the Asthma Quality of Life Questionnaire-Standard (AQLQ-S) overall. Among sub-domains, all domains had improved significantly except emotional function. The result is comparable to previous studies. Baig et al. in their double-blind trial compared montelukast with placebo. They found overall significant quality of life improvement, like our study; however, in their study only the environmental stimuli sub-domain of AQLQ-S had significant improvement compared to placebo [8]. Studies have suggested that montelukast, when given as an add-on therapy with ICS, can significantly improve QOL in asthma patients [9]. Other studies have also reported similar results, especially a study conducted by Virchow and his colleagues, who reported better morbidity as explained by mini-AQLQ scores, when montelukast was used as an adjunct to LABA and ICS [10]. Moreover, the same researcher in his other study investigated the efficacy of 10 mg montelukast in adults with asthma in a phase IV clinical trial, conducted in different centres, and reported that there was an improvement in symptomatology of asthma. Around 92% of patients were satisfied with montelukast and considered continuing montelukast treatment over other asthma medications, and 85% of patients suffering from asthma marked “very good" or "good” in the questionnaire [11].

In an experimental, double-blind, crossover study, researchers gave 20 mg of montelukast two times a day for almost a week to a group of patients and compared the results with the patients taking placebo. They reported that the symptoms produced by leukotriene E4 (LTE4) were significantly improved in the patients taking montelukast [12]. This study resonates with the findings of another research by Bozek et al. who concluded that montelukast when added to a short-acting beta-agonist (SABA) resulted in fewer attacks of exacerbations of asthma [13]. However, there have been contrasting studies as well, which report the opposite. Columbo showed that there was insignificant improvement in asthma with montelukast when given to elderly patients after comparing Asthma Control Test (ACT) scores and measuring biochemical assay at the fourth and eighth week of therapy [14].

Nevertheless, there are various studies that support montelukast in the management of acute exacerbation of asthma as well as chronic asthma of varying severity. Montelukast ameliorates inflammation of the respiratory tract, as well as improves the overall symptomatology of the asthmatic patients. From various data available and our research on this topic, we can safely conclude that pulmonologists should consider using montelukast in the management of asthma as an add-on therapy with ICS or LABA to improve the overall morbidity and quality of life in asthma patients. Other studies should be done to compare if montelukast can replace ICS or not, as ICS has some long term side-effects. More studies are required to study the effect of montelukast on the mortality of asthma patients.

## Conclusions

In this study, there was significant improvement in overall Asthma Quality of Life Questionnaire-Standard (AQLQ-S). Among sub-domains, environment functions, symptoms, and activity limitations had significant improvement. It is important to consider the impact on quality of life of various treatment options available for asthma. Our study, along with various global studies, shows that montelukast is effective in improving patients quality of life.
